# Recurrent ectopic pregnancy on tubal remnant treated by laparoscopic resection: loop and stitch

**DOI:** 10.52054/FVVO.13.2.020

**Published:** 2021-06-28

**Authors:** S Restaino, A Vidiri, L Anchora Pedone, A Finelli, M Distefano, G Scambia

**Affiliations:** Division of gynecological oncology, Department of Obstetrics and Gynaecology, Catholic University of Sacred Heart, L.go A. Gemelli; 00167 Rome, Italy; Catholic University of Sacred Heart, L.go Francesco Vito; 00167, Rome, Italy; Department of Medicine and Aging Sciences University of Chieti-Pescara, Via dei Vestini 131, 66100 Chieti, Italy; Division of gynecological Oncology, Department of Obstetrics and Gynaecology, Catholic University of Sacred Heart, L.go A. Gemelli; 00167 Rome, Italy

**Keywords:** ectopic pregnancy, laparoscopy, endoloops ®

## Abstract

Ectopic pregnancies occur in about 1-2 % of all pregnancies, with a high rate of maternal mortality due to bleeding caused by the rupture of the ectopic pregnancy. Ipsilateral ectopic pregnancy on a tubal remnant after salpingectomy is rare and it is associated with a higher mortality rate when compared to other ectopic pregnancies. Diagnosis and treatment of these pregnancies can be difficult, requiring a multidisciplinary management to plan the best treatment for the patient. The objective of this video is to show the laparoscopic removal of a tubal pregnancy on the stump of a previous salpingectomy with the application of three laparoscopic rings/endoloops ® to isolate the tubal portion from the uterus.

## Introduction

Ectopic pregnancies occurs in about 1-2 % of all pregnancies, with a high rate of maternal mortality due to massive bleeding caused by the rupture of the ectopic pregnancy (Farquhar, 2005). Ipsilateral ectopic pregnancy on a tubal remnant after salpingectomy is rare and it is associated with mortality rates higher than other ectopic pregnancies (Al-Inizi, 2015; Lau and Tulandi, 1999); this is probably due to the poor ability of the remnant portion of the tube to distend as well as the increased vascularity of the area (Takeda et al., 2006). Therefore, this type of ectopic pregnancies requires a multidisciplinary management that allows to plan the right treatment for the patient, taking into account the high risk of bleeding and the risk of rupture of the uterus. We present a case of recurrent ectopic pregnancy on a tubal remnant after ipsilateral salpingectomy treated by laparoscopic resection using for the first time in literature using the Endoloop ligature. We have obtained written consent from the patient to present the case.

## Case report

A 38-year-old, gravida 4, nulliparous woman, with two spontaneous miscarriages and a previous right salpingectomy for a tubal pregnancy, was referred to our hospital at 4+5 weeks of gestation with pelvic pain and spotting. Her serum beta-human chorionic gonadotropin (β-HCG) was 2732; transvaginal ultrasonography revealed the absence of an intrauterine pregnancy and the presence in the right remnant tube of a yolk sac and an embryo pole of 4 mm plus free fluid in the Pouch of Douglas. After a discussion between the gynaecological surgeon and the sonographer, we fully discussed the possible treatment options with the patient. Considering the β-HCG level, the clinical symptoms and the absence of an emergency, after obtained informed consent, a laparoscopy was performed with removal of the right tubal remnant and the pregnancy, using the Endoloop ligature (ENDOLOOP ® Ligature) and myometrial stitch.

## Procedure

The patient, under general anaesthesia, was positioned in the dorsal lithotomy position with both legs supported in stirrups with a Trendelenburg tilt and arms alongside the body. Four sterile trocars were used. A 12 mm port was inserted at the umbilicus for the telescope (0° high-definition telescope). Three additional 5 mm ports were placed under direct vision after the pneumoperitoneum was achieved. Two trocars were inserted in the right and left lower abdomen medial to the right obliterated umbilical artery and lateral to the inferior epigastric vessels at 1.5 cm above the anterior superior iliac spine. One further 5-mm trocar was inserted along the midline at least 8 cm from the umbilicus. The intervention can be divided into 4 consequential phases.

**Figure 1:**
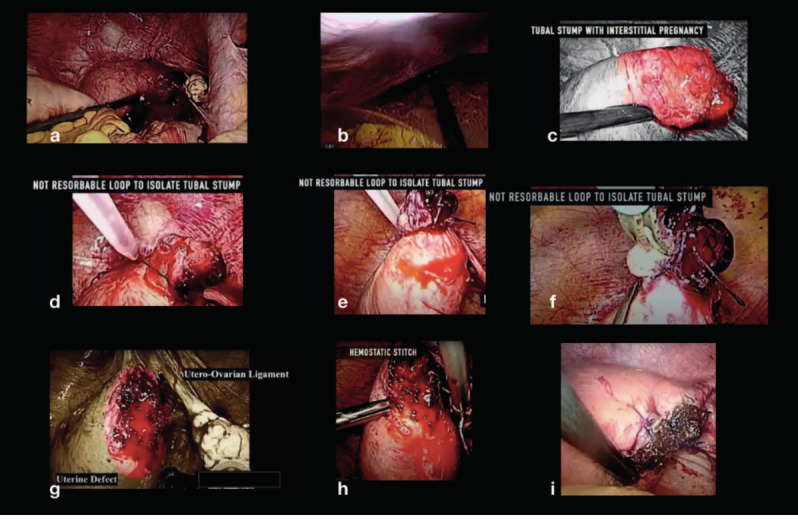
— Step by step procedure.

1. Evaluation and Cleaning: toilet of the pelvis and upper abdomen from the blood.
2. Adhesiolysis: lysis of the adhesions present so as to isolate, and better characterise the ectopic pregnancy. (Figure 1 a-c)
3. Development: subsequent apposition of three Endoloops ® (two proximal and one distal to the uterus) and removal of the pregnancy in its entirety. This allows a better haemostatic control at uterus level and avoids possible loss of gestational tissue into the pelvis. (Figure 1 d-f)
4. Reconstruction: Four independent laparoscopic points are applied, after removing the Endoloops®, to reconstruct the myometrium and achieve hemostasis. (Figure 1 g,i)
5. Extraction: the tubal renmant and the ectopic pregnancy are extracted using an endobag.

The post-surgical course was uneventful. The total operation lasted 65 min and reported blood lost was approximately 50 cc. The patient had a normal post-operative recovery with an average VAS score of 3, and was discharged about 24 hours after surgery. The results of the histological exam was: "chorionic tissue in the remnant tube".

## Discussion

In the literature, the terms “angular”, “interstitial” and “cornual” have often been exchanged inappropriately, but the 2014 edition of Williams’ Obstetrics and more recently the ESHRE working group on Ectopic Pregnancy, differentiates them precisely (Ectopic Pregnancy, 2019; Kirk et al., 2020). An interstitial pregnancy is an extrauterine pregnancy, resulting when implantation occurs within the myometrial fallopian tube or interstitium. They are generally considered non-viable and their mortality rate approaches 2.5% due to the increased risk of uterine rupture and severe haemorrhage, which generally occur at over 12 weeks of gestation (Moawad et al., 2010). A cornual pregnancy historically describes the implantation of the intrauterine fundus within the bicornuate or abnormal septate uterus, while an angular pregnancy is an eccentric intrauterine pregnancy with implantation of the embryo in the upper lateral corner of the uterine cavity, and should be considered paraphysiological and vital. Differentiating interstitial from angular pregnancies has proven difficult despite advances of 3D sonography and MRI.A 2017 retrospective review of 44 interstitial and angular pregnancies found that the myometrial thickness <5 mm was present in 96% of interstitial pregnancies but also 50% of angular pregnancies (range 3–8 mm); an “interstitial line sign” (an echogenic line connecting the endometrial stripe to an interstitial gestational sac) appears to be more specific for differentiating the interstitial pregnancy with reported rates of 100% specificity and 80% sensitivity (Grant et al., 2017). Obstetricians must maintain vigilance in considering the possibility of angular pregnancy in the differential diagnosis of any pregnancy suspected to be interstitial.

Diagnosis of these pregnancies can be difficult and the concern for disrupting an intrauterine pregnancy may cause hesitation during evaluation and management. Ipsilateral ectopic pregnancy in a tubal remnant after salpingectomy is rare; the exact incidence of an ectopic pregnancy in the tubal remnant following salpingectomy is not currently known, although Takeda et al. (2006) reported an incidence of 1.16 % in their department from January 1994 to August 2005. This type of ectopic pregnancy is associated with mortality rates higher than other ectopic pregnancies, requiring a multidisciplinary management to plan the right treatment for the patient.

The treatment of these conditions depends on the gestational age, the values of β-hCG levels, ultrasound features, clinical presentation and patient preference. It may require a medical, surgical or close observation approach. In case of early diagnosis and a low value of β-hCG, medical therapy with methotrexate is feasible. Surgical management of an ectopic pregnancy is required when a patient presents with haemodynamic instability, symptoms of an ongoing ruptured ectopic mass or signs of intraperitoneal bleeding.

## Conclusion

It was the first case, in our structure, of an interstitial pregnancy treatment with this method. The post- surgical course was uneventful and the woman was discharged without any complications. In our case, the aim was to show how to manage an ectopic pregnancy on a previously removed salpinx using the laparoscopic approach. Furthermore, this case highlights how the use of laparoscopic endoloops should always be evaluated to reduce intraoperative bleeding. We believe that this approach is effective and an a safe alternative to manage this type of gynaecological pathology. monofilament suture and by avoiding the complete passage of the needle through the vaginal wall during suturing. So far there is no consensus what type of suture material should be used for the colporrhaphy, as it is clear that a nonabsorbable suture for the apical suspension leads to better results (Bergman et al., 2016; Zebede et al., 2013). Absorbable sutures can be used for the colporrhaphy, as been highlighted by Noé with the slow absorbable type being associated with less risk of symptomatic recurrence (Noé et al., 2019; Bergman et al., 2016).

The modifications of the conventional surgical techniques, that we demonstrate in our video, a pectopexy and a sacrocolpopexy with native tissues fascia repair, are inspired by potential need of the gynaecologist to search for an alternative way to manage patients with POP without using a synthetic prothesis. With our video we present a feasible and reproducible approach using only sutures for the suspension of the cervix and the vagina. Advanced skills in laparoscopic suturing and excellent knowledge of the pelvic anatomy are the prerequisites to perform the corresponding techniques. The data from studies examining these new concept mesh-less techniques show promising short-term results with low complication rates compared to the procedures using prothesis. Further studies are needed to evaluate and determine the optimal way of management of patients with apical POP using a mesh-less approach.
